# The Molecular Model of Organic Matter in Coal-Measure Shale: Structure Construction and Evaluation Based on Experimental Characterization

**DOI:** 10.3390/molecules28135203

**Published:** 2023-07-04

**Authors:** Kunjie Li, Hongwu Tian, Yanxia Liang, Wei Guo, Yuqiong Zhao, Yanjun Meng, Shaoqi Kong

**Affiliations:** 1 Key Laboratory of Coal Science and Technology (Ministry of Education), Taiyuan University of Technology, Taiyuan 030024, China; likunjie@huaxingas.com (K.L.); zhaoyuqiong@tyut.edu.cn (Y.Z.); 2 Shanxi Huaxin Gas Energy Institute Co., Ltd., Taiyuan 030032, China; 3 National Engineering Research Center of Intelligent Equipment for Agriculture, Beijing 100097, China; tianhw@nercita.org.cn; 4 Department of Agricultural Electrification and Automation, Shanxi Agricultural University, Jinzhong 030801, China; bnn@sxau.edu.cn; 5 College of Mining Engineering, Taiyuan University of Technology, Taiyuan 030024, China; mengyanjun@tyut.edu.cn

**Keywords:** organic matters, shale, macromolecular model, pores

## Abstract

To investigate the molecular structure and micropore structure of organic matters in coal-measure shale, the black shale samples of the Shanxi formation were collected from Xishan Coalfield, Taiyuan, and a hybrid experimental–simulation method was used for realistic macromolecular models of organic matter (OM). Four experimental techniques were used to determine the structural information of OM, including elemental analysis, state ^13^C nuclear magnetic resonance (^13^CNMR), X-ray photoelectron spectroscopy (XPS), and Fourier transform infrared spectroscopy (FTIR). With structural parameters, two-dimensional (2D) average molecular models of OM were established as C_177_H_160_O_8_N_2_S with a molar weight of 2474, which agreed well with the experimental ^13^C-NMR spectra. A realistic three-dimensional (3D) OM macromolecular model was also reconstructed, containing 20 2D molecules with a density of 1.41 g/cm^3^. To determine the connectivity and spatial disposition of the OM pores, focused ion beam microscope (FIB-SEM) and transmission electron micrographs (TEM) were utilized. The 3D OM pores models were developed. The results show that whether the OM pores varied from 20 to 350 nm as obtained from FIB-SEM images or less than 10 nm as observed in the TEM images, both were of poor connectivity. However, the ultra-micro pores from the 3D OM macromolecular model varied from 3Å to 10 Å and showed certain connectivity, which may be the main channel of diffusion. Furthermore, with the pressure increased, the methane adsorption capacity of the 3D OM model increased with a maximum value of 103 cm^3^/g at 7 MPa, indicating that OM pores less than 1 nm have a huge methane adsorption capacity. Therefore, our work provides an analysis method that is a powerful and superior tool in further research on gas migration.

## 1. Introduction

In the year of 2021, shale gas production in China increased to 26 trillion cubic meters, which accounts for more than 10% of the total natural gas production [1]. In the next five years, shale gas production will continue to increase rapidly and could contribute more than 20% of the total natural gas production [2,3]. China is rich in coal resources, and coal-measure strata are widely distributed, including in Shanxi, Shaanxi, Xinjiang, and Inner Mongolia [4]. As the main component of coal-measure strata, shale (mudstone and siltstone) develops with coal, sandstone and limestone, and its total thickness can reach hundreds of meters. In some layers, the dark shale develops very well, with a total organic carbon content (TOC) up to 30%, showing good hydrocarbon generation capacity [5]. It is estimated that the exploitable resources of coal-measure shale gas measure 8.97 × 10^12^ m^3^, which promises great potential for resource exploration [4,5,6]. For coal-measure shale, its single layer is generally less than 20 m in thickness, and it is thus more suitable for co-exploitation with coal-bed methane or tight sandstone than for single mining.

In the coal-measure strata, carbonaceous mudstone is often developed near the coal seam, especially in the roof and floor of the coal seams. The TOC of carbonaceous mudstone is generally more than 10%, which plays a key role in the development of OM pores [7]. As the thermal maturity increases, numerous nano-pores are generated in the OM, which could provide larger surface areas and higher sorption energy for gas adsorption. It has been found that the methane adsorption capacity of shale is positively correlated with TOC. Methane molecules are adsorbed on the surface of OM molecules in the pores of shale, and OM pores are essential for shale gas storage [7,8,9,10]. Thus far, OM pores have been characterized with multiple measurements, such as field emission-scanning electron microscopy (FE-SEM), transmission electron microscopy (TEM), X-ray computed tomography (X-CT), low-pressure N_2_ and CO_2_ gas adsorption, etc. The OM pores were determined to be silt-like, ellipse-shaped, round, and irregular, etc., all of which can be classified into three categories: micropores (<2 nm), mesopores (2–50 nm), and macropores (>50 nm) [11,12,13]. Previous studies based on N_2_ and CO_2_ adsorption analysis have shown that most OM pores are less than 10 nm and are dominated by 2–4 nm [10]. However, limited by the device resolution, only the pores more than 10 nm could be identified, while the distribution and morphological characteristics of other pores (less than 10 nm) could not be directly observed [14,15,16]. The OM pores (less than 10 nm) can only be studied by computer modeling. Moreover, with the computational macromolecular model, many aspects related to coal or OM can be studied, such as the distribution features of micropores; characteristics for gas adsorption, desorption, and diffusion; mechanisms of thermal structural evolution and reaction, etc. [17,18,19,20].

The microstructure of OM pores is essential and is composed of macromolecules and their fragments. Therefore, it is necessary to first construct a macromolecular model of OM to study the nanopore structure of OM. A number of advanced analytical techniques have been used to determine the chemical structure and molecular composition of OM, such as X-ray diffraction (XRD), XPS, FT-IR, ^13^CNMR, and high-resolution transmission electron micrographs (HRTEM) [21,22,23]. With these methods, the distribution and occurrence information of the carbon skeleton, functional groups, and heteroatoms of coal or OM can be determined. There are two main ways to construct coal or OM 3D macromolecular models currently. One approach is to construct the average 2D OM molecular structure using the information of macromolecular composition as determined by element analysis, XPS, FTIR, and ^13^CNMR. Then, the 3D models are generated by molecular modeling and are optimized by cooperation with the experimental data of physical density and microporosity [24]. The other way is to construct 3D macromolecular models directly using the parameters of aromatic structures, aliphatic side chains, and heteroatoms obtained from HRTEM, ^13^CNMR, and XPS. The models are validated and modified using the data of physical structural properties such as bulk density, pore size distribution, etc. [21,25,26].

In this work, microscopic detection techniques and computer simulation methods were combined to investigate the structural characteristics of OM in coal-measure shale. The chemical-structural properties of OM extracted from Shanxi formation shale were determined with ^13^CNMR, FTIR, and XPS techniques. The micropore structure of OM was characterized with low-pressure CO_2_ adsorption, FIB-SEM and TEM. A realistic 3D OM model was established based on the average 2D model and was optimized and validated by the experimental data of bulk density and CO_2_ adsorption. Furthermore, the 3D OM pore model was reconstructed with FIB-SEM images, and the diffusion mechanism of methane in OM was discussed by analyzing the distribution and connectivity of pores as well. The methane adsorption of the 3D OM macromolecular structure was studied, and its capacity was evaluated.

## 2. Result and Discussion

### 2.1. Elemental Analysis, ^13^CNMR, and FTIR Data

The results of the elemental analysis for the OM sample (Table 1) show that the content of C is 87.61 wt%, while the H is relatively low at only 3.71 wt%, suggesting the feature of type III kerogen. The O occupies 5.51 wt% of the OM sample, which is a not high content, indicating that the OM is at a highly mature stage. The helium density of the OM sample is 1.40–1.42 g/cm^3^.

**Figure 1:**
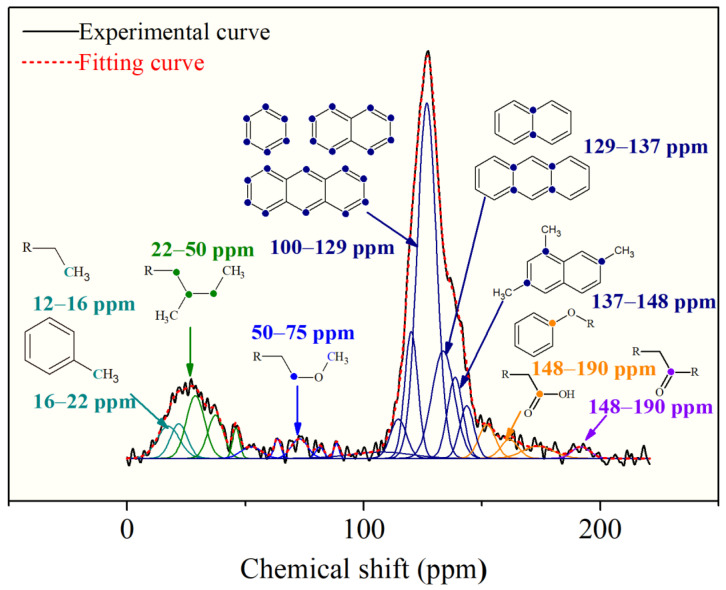
Peak-fitting curves of the ^13^CNMR spectrum with wavenumber assignment.

The ^13^CNMR and FTIR data can be used to determine the chemical structure of the macromolecule for OM reconstruction. The ^13^CNMR spectrum of the OM sample is shown in Figure 1. There are three peak regions in the ^13^CNMR spectrum: the main peak is in 100–150 ppm and the other two median peaks in 0–50 ppm and 180–220 ppm, respectively. The largest peak in 100–150 ppm is assigned to the chemical shifts of aromatic carbons region. Peaks distributed in the regions of 15–22 ppm and 22–50 ppm correspond to aliphatic –CH_3_ and –CH_2_, respectively. The peaks in the chemical shift range of 180–210 ppm are of median intensity [21,24], which arises from aliphatic carbons connecting with carbonyl oxygen. Some small peaks located in 50–90 ppm are aliphatic carbons connecting carbonyl carbon to the hydroxyl carbon.

To quantify the structural parameters, the ^13^CNMR spectra were further treated by peak fitting with Origin 9.0. The splitting peak position of each functional group and its relative percentage are shown in the Table 2. The skeleton structure parameters of OM were obtained. Moreover, the average ratios of aromatic bridge carbon to aromatic peripheral carbon (*X_BP_*) were calculated by the following equation:

*X_BP_* = *f_a_^B^*/(*f_a_^S^* + *f_a_^H^* + *f_a_^P^*) (1)

*X_BP_* is calculated to be 0.195 for OM samples, which is lower than coal in the same layer.

**Figure 2:**
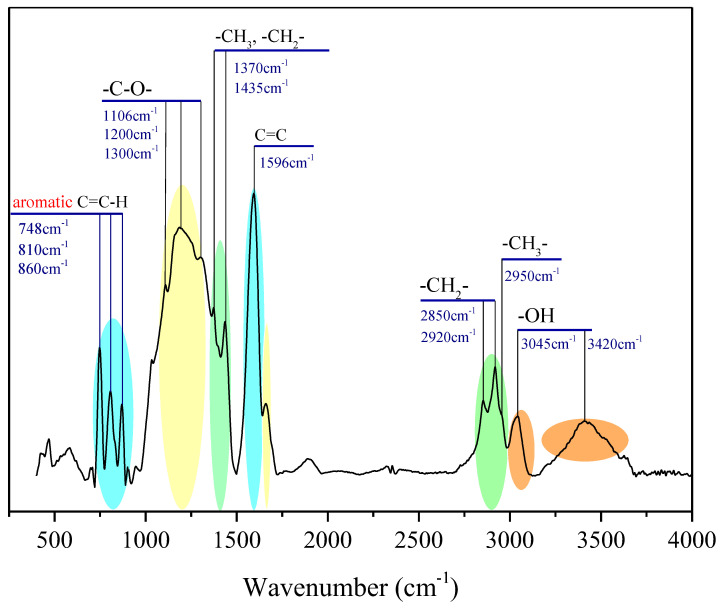
The FTIR profile of the OM sample with wavenumber assignment.

Figure 2 shows the FTIR spectra of the OM sample, which were corrected by the baseline. According to the previous studies, the identified absorbance bands were classified into four regions: the aromatic structure region (900–700 cm^−1^), oxygen functions’ region (1800~1000 cm^−1^), the aliphatic structure (3000–2800 cm^−1^), and hydroxyl structure region (3600–3000 cm^−1^) [27,28].

The peaks at 900–700 cm^−1^ are due to out-of-plane bending of the aromatic C–H, and the peak at 748 cm^−1^ is of the highest intensity in this region, indicating the di-substitution of the benzene ring is dominant. The peaks of 1110–1330 cm^−1^ represent the C–O of the oxygen-containing functional groups, such as ethers, hydroxyls, etc. The prominent peaks on the spectra are located at the range of 1370–1450 cm^−1^ and 2850–2950 cm^−1^, which are both assigned to aliphatic chains [27,29], suggesting a certain amount of oxygen-containing functional groups and aliphatic chains exist in the OM samples. The peak near 1600 cm^−1^ is obvious and sharp, which further confirms the existence in large quantities of aromatic carbons in the OM chemical structure.

**Figure 3:**
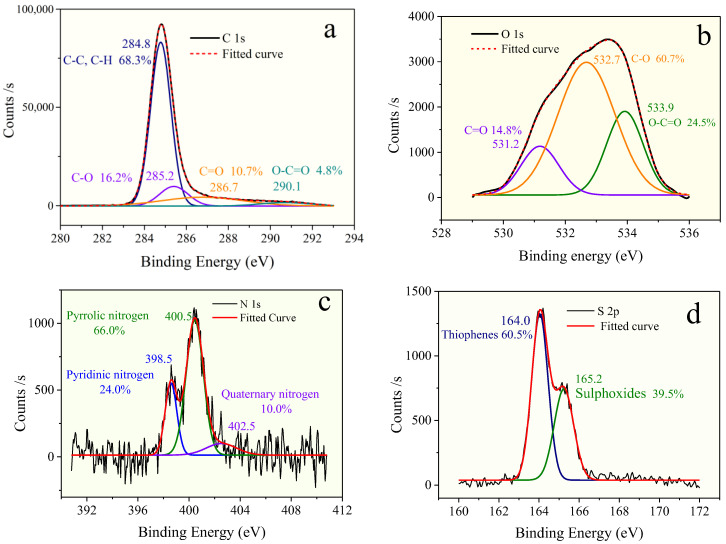
XPS: (**a**) C 1s scan, (**b**) O 1s scan, (**c**) N 1s scan, and (**d**) S 2p scan spectra of OM in the Shanxi formation shale.

The chemical-functional groups on the surface of OM were measured by XPS experiment. The type and distribution of O, N, and S heteroatoms in OM were determined by peak fitting of XPS spectra [30]. The results are shown in Figure 3, and the distribution of the corresponding heteroatom functional groups is listed in Table 3.

The deconvolution of the C 1s signal generated two peaks at binding energies of 284.8 eV, 285.2 eV, 286.7 eV, and 290.1 eV, which correspond to the groups of C–C or C–H (68.3%), C–O (alcohol, phenol, or ether; 16.2%), C=O (carbonyl; 10.7%), and COOH (carboxyl; 4.8%), respectively [31]. As Figure 3 and Table 3 show, the form of oxygen existing in the OM sample is mostly C–O (60.7%), followed by C=O (24.5%), and the least is COOH (14.8%).

The signal-to-noise ratios of XPS of N 1s and S 2p spectra did not require smoothing processing before peak fitting. The result of deconvolution of the N 1s signal (Table 3) suggests that the existence of nitrogen consists of three types, including pyridine (25.1%), pyrrolic (68.0%), and quaternary nitrogen (6.9%), and nitrogen atoms in the OM samples are mainly in the formation of pyrrolic nitrogen.

Based on the quantitative analysis of S 2p spectra, as shown in Figure 3 and Table 3, sulfur atoms in the OM are in two formations: thiophenes sulfur and sulphoxides sulfur, which contribute 66.5% and 39.5% of the total sulfur content, respectively.

### 2.2. Construction of 2D Molecular Model

The condensed aromatic nuclei in OM are similar to those of coal, which consist of aromatic benzene rings, aliphatic rings, hydroaromatic rings, and aromatic heterocyclic structures (nitrogen, sulfur, etc.) [18,32]. The ratio of aromatic bridge carbon/aromatic weekly carbon (*X_BP_*) of OM samples is 0.196. Combined with the value of the carbon content, the average aromatic ring number of the OM sample is two, and the aromatic structural units of OM are mainly composed of naphthalene, benzene, anthracene, pyrene, etc. Generally, the OM molecules with high maturity have the relative molecular weight of about 3000, and the number of carbon atoms is nearly 200 [33]. According to the experimental ^13^CNMR data, the types and numbers of aromatic structures in OM macromolecular were determined and are given in Table 4. The number of aromatic carbon atoms is 134. The *X_BP_* of the OM single-molecular model is 0.195, which is close to the experimental result.

The aliphatic structure in OM includes side chains, bridge bonds, saturated rings, hydroaromatic rings, etc. As shown in Table 1, the aromaticity fa’ value of OM is 0.76. The atomic numbers of total carbon and aliphatic carbon are 177 and 43, respectively. The aliphatic structure in OM includes side chains, bridge bonds, saturated rings, hydroaromatic rings, etc. The ratio of f_al_^*^:*f_al_^H^*:*fa^O^* is about 6:10:4, which indicates the main existence of the aliphatic structure is hypomethyl carbon or quaternary carbon, with a certain amount of methyl carbons and oxygen lignant carbon.

The result of elemental analysis shows that the atomic ratios of O/C, N/C, and S/C of the OM samples are 0.047, 0.012, and 0.009 (Table 1), respectively. Combined with the XPS result, eight oxygen atoms are presented in the model. The number of ether (C–O) groups, carbonyl groups (C=O), and carboxyl groups (O–C=O) is 12, 3, and 5, respectively. Furthermore, the nitrogen and sulfur content values in the OM model are relatively low. There are two nitrogen atoms in the forms of pyrrole, and only one sulfur atom is present in thiophene in the OM single-molecular structure (Table 3).

**Figure 4:**
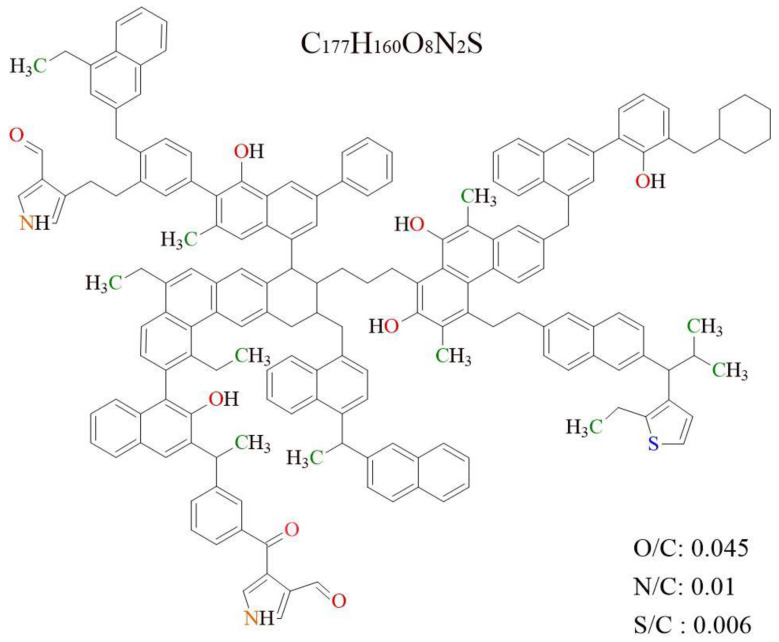
The 2D OM molecular model in Shanxi formation shale.

**Figure 5:**
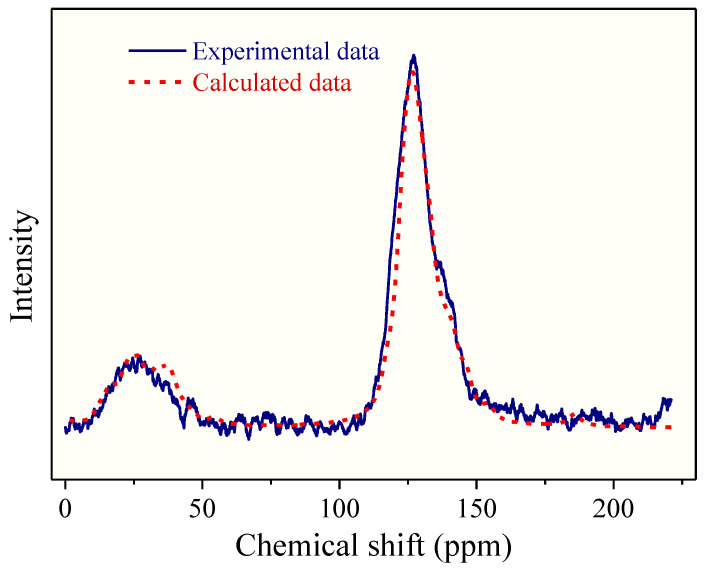
Comparison between the experimental ^13^CNMR spectrum and the calculated ^13^CNMR spectrum based on the reconstructed 2D OM molecule model in Figure 4.

Based on the experimental data of FTIR, ^13^CNMR, and XPS, the 2D chemical structure of the Shanxi formation shale’s OM was established, as shown in Figure 4. The molecular formula of the constructed OM single model is C_177_H_160_N_2_O_8_S, and its total molecular weight is 2474. The atomic ratios of O/C, N/C, and S/C of the OM single-molecular model are 0.045, 0.011, and 0.006, respectively, which is close to the experimental data. The ^13^CNMR data of the estimated OM model were compared with the experimental data in Figure 5. The simulated ^13^CNMR spectrum fits well with the experimental spectra. It is noteworthy that the molecular model is a statistical average structure of OM since it is obtained based on the statistical average results.

### 2.3. Construction of 3D Macromolecular Model of OM

Based on geometric optimization of the single 2D OM molecular model, the three-dimensional macromolecule model was reconstructed, combined with helium density experimental data. The validation of the constructed 3D OM model was further checked by the CO_2_ adsorption data. The number of 2D OM molecules in the 3D model and the dimension of the unit cell can be estimated as follows:

D = N × M/(L^3^ × N_A_)

where D is the lattice density, g/cm^3^, N is the number of 2D OM molecules, M is the single 2D OM molecular weight; L is the side length of the lattice, nm; and N_A_ is the Avogadro constant, 6.02 × 10^23^.

Moreover, the density of the 3D OM macromolecular model can be calculated as the following:

D_He_ = N × M/(L^3^ − V_He_) × N_A_)

D_He_ is helium density, g/cm^3^; and V_He_ is the free volume using helium as the probe (radius = 0.13 nm), nm^3^.

**Figure 6:**
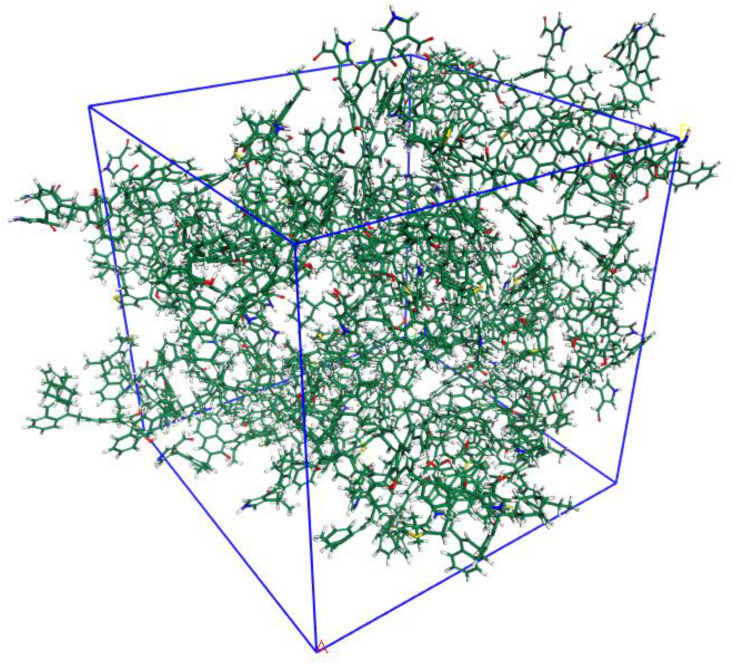
Three-dimensional OM macromolecular model (C_3540_ H_3200_ O_160_ N_40_ S_20_) composed of 6960 atoms in a periodic cubic box (43.5 Å × 43.5Å× 43.5 Å).

The 3D OM macromolecular model is shown Figure 6. The unit cell is the cubic type with a lattice size of 4.35 nm, which consists of 20 OM molecules and contains 6960 atoms (C_3540_ H_3200_ N_40_O_160_ S_20_), with a total molecular weight of 49,480.

**Figure 7:**
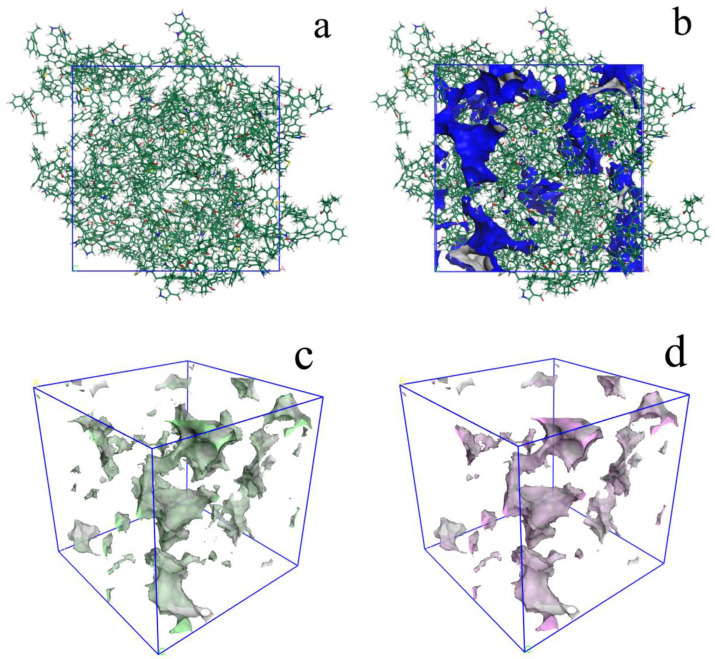
Micropores in the 3D OM macromolecular model (Probe: CO_2_): (**a**) 3D macromolecular model; (**b**) micropores (<1 nm) in 3D macromolecular model; (**c**) micropores in 3D macromolecular model; (**d**) accessible micropores in 3D macromolecular model.

The calculated D_He_ of unit cell is 1.41 g/cm^3^, which agrees with the experimental result of 1.40–1.42 g/cm^3^. The micropore volume of the 3D OM model (Figure 7), calculated using Poreblazer with CO_2_ as the probe, was 0.0308 cm^3^/g. The micropore volume (pore size less than 1.1 nm) measured by the CO_2_ adsorption test is 0.0269 cm^3^/g (Figure 7c,d). The estimated model value is larger than the experimental data in terms of micropores’ volume.

Based on 3D OM macromolecular model, the ultramicropores (less than 1 nm) were reconstructed using CO_2_ as the probe. These pores are composed of accessible pores and inaccessible pores (Figure 7c,d). The former are open pores, and these can be entered from outside. The latter are closed by the OM molecules. The Figure 7 indicates that the accessible pores are connected in some local space. Thus, these fine, connected pores should be the channels for gas migration during the maturation and hydrocarbon generation of OM.

**Figure 8:**
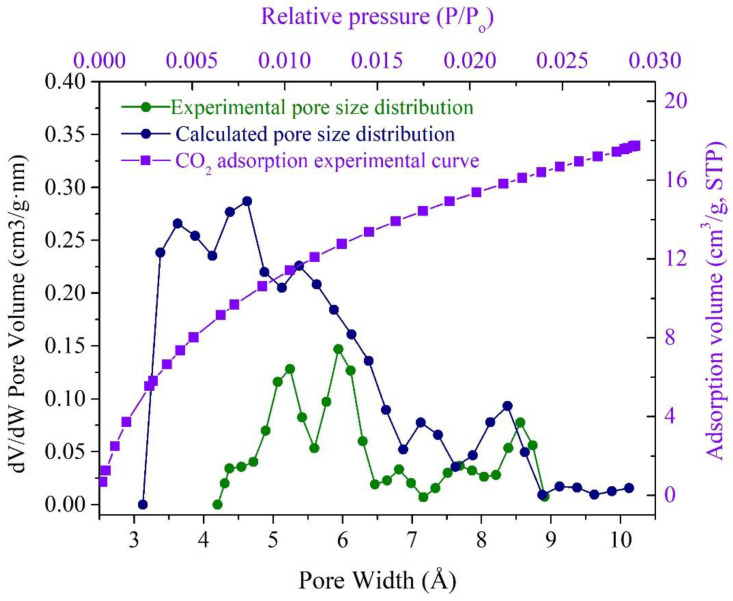
Comparison of PSDs estimated from the CO_2_ adsorption and calculated from the 3D OM macromolecular model.

Figure 8 shows the pore size distribution (PSD) obtained by the CO_2_ adsorption experiment and calculated from the 3D OM macromolecular model using CO_2_ as the probe. The shapes of the two curves are very similar with each other and in the range of 4–9 Å. Compared with the calculated results, the experimental result has a narrower size range and weaker PSD peaks. The calculated pore size is within the range of 3.3–10 Å and mainly located between 3 and 7 Å. However, the experimental PSDS was in the range of 4–9 Å and without the data from 3 to 4 Å. This is possible because the pores between 3–4 Å are close to CO_2_ molecules (3.3 Å), and it is difficult to identify this by the CO_2_ adsorption experiments. Moreover, the calculated result contains a certain number of inaccessible pores. Based on the above reasons, the experimental pore volume (0.0269 cm^3^/g) is smaller than the calculated value of the 3D OM macromolecular model (0.0308 cm^3^/g).

### 2.4. Pore Structure

#### OM Pores in the FIB-SEM/HREM Images

**Figure 9:**
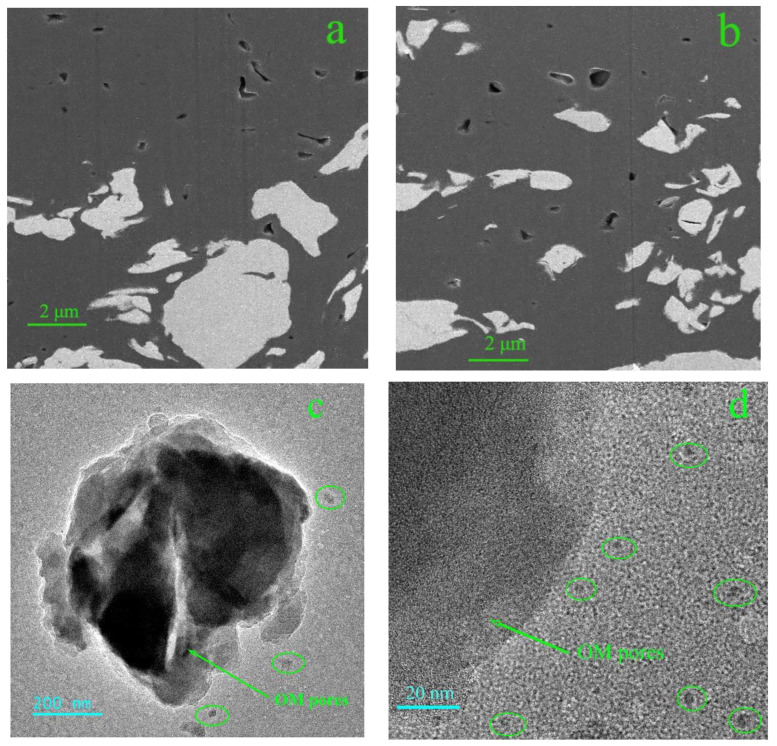
(**a**,**b**) OM pores in the FIB-SEM images; (**c**,**d**) OM pores in the TEM images.

The observation results of the FIB-SEM and TEM (Figure 9) show that a large number of OM nanopores are well developed, with a size from several nanometers to tens of nanometers. The OM pores are mostly isolated pores with poor connectivity, which exhibit various shapes from ellipsoid to elongated bubble and irregularly polygon, and ellipsoid is the most common shape (Figure 9a,b).

The distribution and connectivity of OM pores are the key factors influencing the gas storage and flow capacity of shale. The TEM images (Figure 9c,d) suggest that OM pores less than 10 nm are isolated and unconnected pores as well, which provides space for the gas storage. However, the problem is in determining how the gas is transported in the OM pores and adsorbed on the surface of the OM.

**Figure 10:**
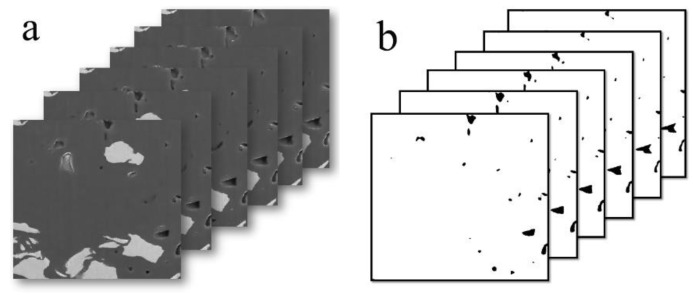

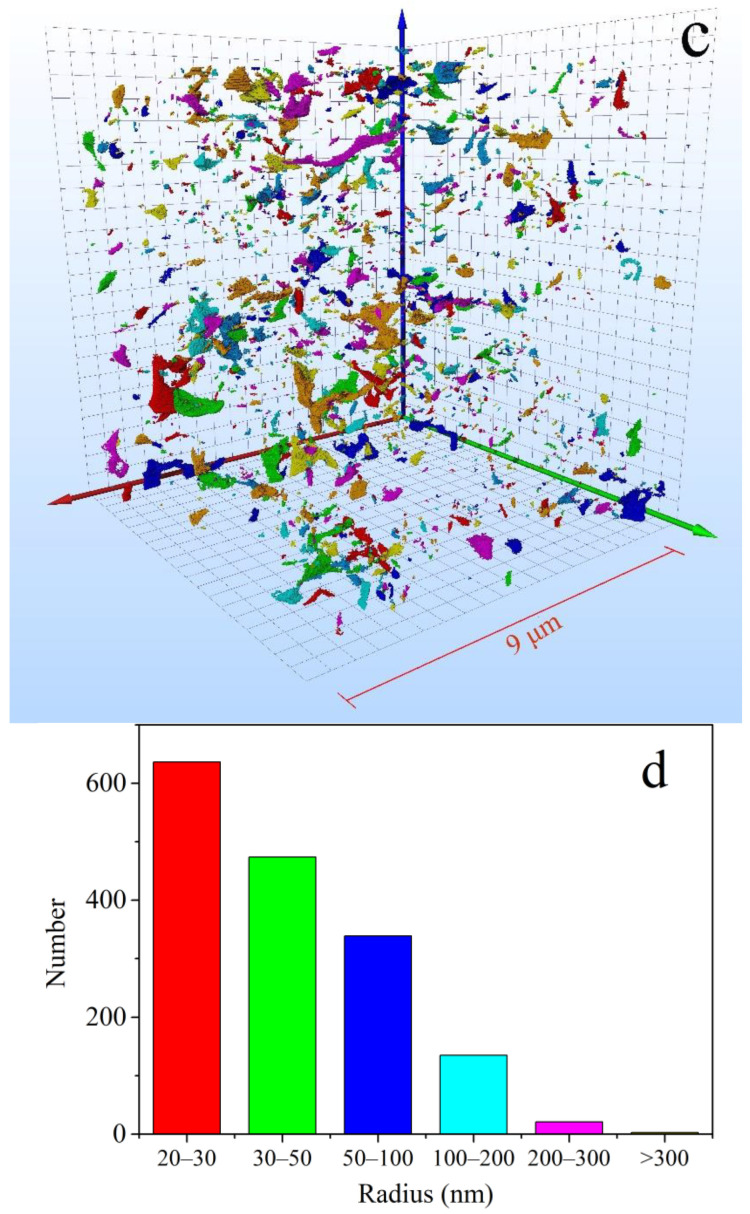
Three-dimensional OM micropores model reconstructed based on sequent FIB-SEM images. (**a**) 2D FIB-SEM images; (**b**) binary FIB-SEM pore image; (**c**) 3D OM micropores model reconstructed based on sequent FIB-SEM images (9μm×9μm×9μm); (**d**) pore size distribution of OM pores in the 3D OM micropores mode.

To investigate the connectivity and spatial disposition of OM pores, a 3D OM pore structure model of the selected area was reconstructed based on the treatment of noise reduction and segmentation on 500 sequential FIB-SEM digital images. The size of the 3D OM pore structure model is 9.0 × 9.0 × 9.0 μm, with a revolution of 20 nm (Figure 10). Statistical analysis indicates that OM pore size varies from 22 nm to 350 nm. As the Figure 10c shows, the OM pores (more than 20 nm) are all isolated pores and are randomly distributed in the shale with poor connectivity.

In the process of shale diagenesis, OM macromolecules condense gradually by the combined effect of temperature and pressure. Aliphatic chains and functional groups are shortened and broken to repel hydrocarbons and inorganic gases, resulting in the presence of a large number of nanopores in the macromolecular structure. Although these numerous pores are very fine in size (less than 1 nm), they can act by connecting larger isolated pores and thus become migration channels for gas molecules.

### 2.5. The Adsorption Capacity of the 3D OM Model

**Figure 11:**
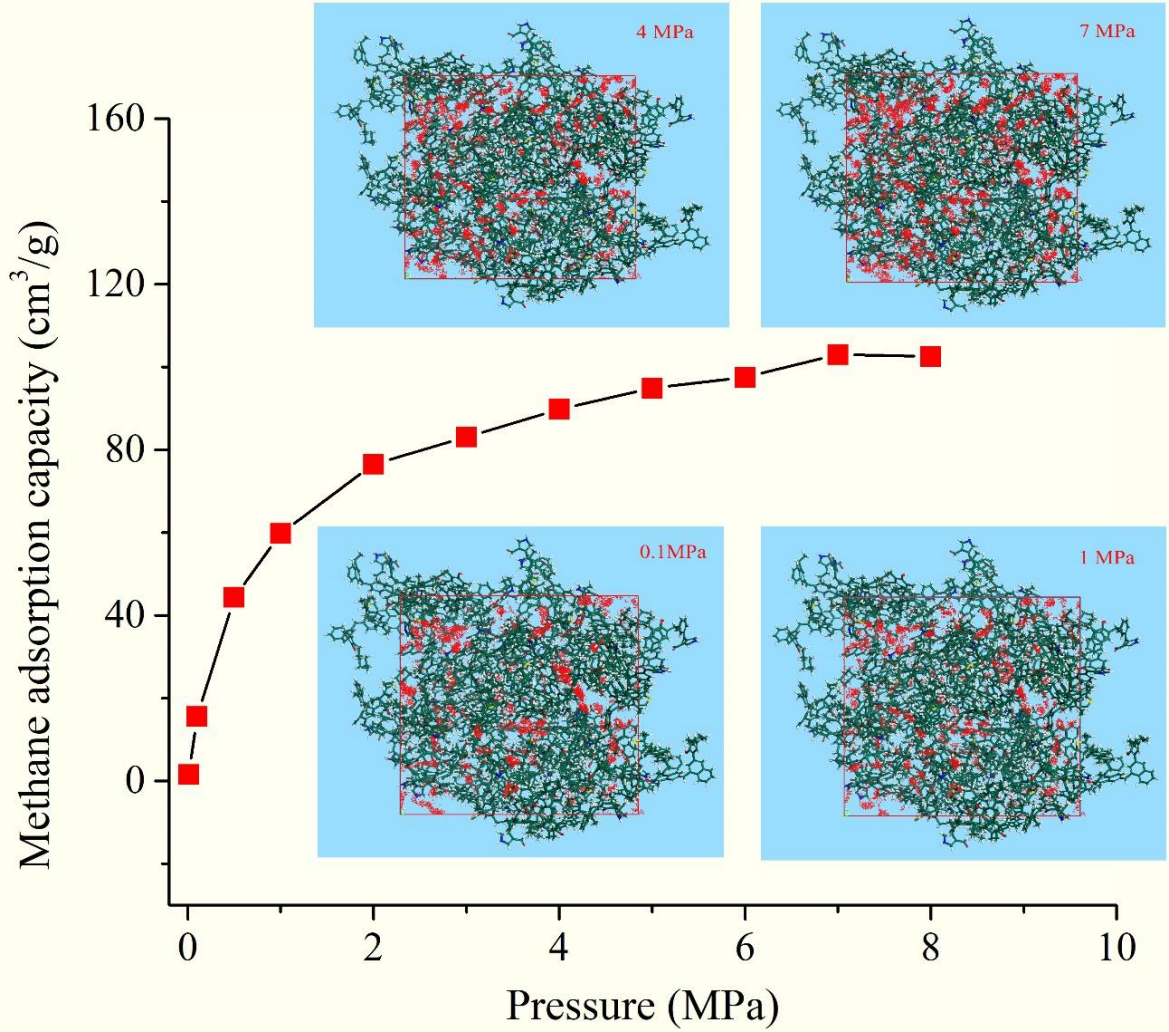
The methane adsorption isotherm of the 3D macromolecular model.

To investigate the methane adsorption capacity of OM micropores, the adsorption quantity of the 3D OM macromolecular model was calculated from 10 kPa up to the final pressure of 8 MPa at 25 °C (Figure 11). In the pressure range of 0–4 MPa, the absolute adsorption of methane increases rapidly from 1.56 cm^3^/g to 89.8 cm^3^/g. When the pressure is greater than 4 MPa, the sorption isotherm increases slowly downward and tends to balance, with the largest value of 103 cm^3^/g at 7 MPa. Considering that the pores in the 3D model are less than 1 nm, methane adsorption in micropores is composed of two forms: gas adsorbed on the surface of pores and gas filling in free volume. The adsorbed phase volume is equal to the pore volume, and the absolute adsorption capacity could be regarded as the adsorption capacity for the pores in the 3D model.

It was calculated that the values of V_L_ and P_L_ are 165 cm^3^/g and 1.56 MPa, respectively, indicating the OM pores (≤1 nm) have a huge methane adsorption capacity and lower desorption pressure.

## 3. Sample and Methods

### 3.1. OM Isolation

Six black shale samples were collected from the Shanxi formation at the roof of NO. 2 coal under the mine in Guandi Coal Mine of Xishan coalfield (Taiyuan, Shanxi, China). Considering the convenience of extracting OM, a representative OM-rich sample (XS-05) with a TOC value of 20.3% was selected as the study object. The other five samples with TOC contents from 3.2% to 4.8% were abandoned (Table 5). The sample XS-05 was firstly crushed into powder with a size less than 74 μm and then was dried at 60 °C for 8 h. Then, demineralization was performed on the sample using a HCl/HF/HCl acid treatment to remove mineral matters, and the OM sample (XS-05D) was obtained by the steps of washing with distilled water and drying until arriving at constant weight [34].

### 3.2. OM Chemical Structure Analysis

The macromolecular structures of the OM sample (XS-05D) were characterized by elemental analysis, ^13^CNMR, XPS, and FTIR.

#### 3.2.1. Elemental Analysis

The elemental analysis was carried out following the international standards ISO 11722: 2013 and ISO 7404–5: 1984. The contents of C, H, N, and S were detected directly on a Germany Vario EL III elemental analyzer, and the O content was obtained by difference calculation.

#### 3.2.2. ^13^CNMR Experiment on the OM

Solid-state ^13^CNMR tests were performed on a Bruker Avance III 600 MHz spectrometer in Institute of Coal Chemistry; Chinese Academy of Sciences, Taiyuan, China, operating at 151.0 MHz with 4 mm MAS probe. CP (cross-polarization) and TOSS (total sideband suppression) techniques were adopted to suppress the sidebands, with a contact time of 5 ms, a magic angle rotation speed of 15 kHz, and a recycle delay time of 3 s.

#### 3.2.3. XPS Experiment on the OM

XPS spectra were generated using an ultra-high vacuum (UHV) system with the surface analysis system (ESCALAB250 Xi, USA) in Taiyuan University of Technology, Taiyuan. The main C1 s peak (284.8 eV) was employed to calibrate the binding energy position. The survey scan was taken at the pass energy of 100 eV with the step size of 1.00 eV, and the narrow high resolution spectra were recorded at the pass energy of 20 eV with the step of 0.05 eV.

#### 3.2.4. FTIR Experiment on the OM

The FTIR measurement of the OM sample was performed using a Bruker Vertex 80v spectrometer in Taiyuan University of Technology, Taiyuan, China. The sample was first mixed with KBr and fully ground in a ratio of 1:200 and then pressed into a slice under 10 MPa for 1 min. The FTIR spectrum was obtained in the frequency range of 4000–400 cm^−1^, with a resolution of 4 cm^−1^.

### 3.3. Characterizing Pore Structure of Samples

The micropore structure of OM was measured with CO_2_ adsorption. The OM pores were characterized according to size, shape, and number by using the FIB-SEM/TEM.

#### 3.3.1. Gas Adsorption and Helium Density Test Experiments

The CO_2_ adsorption isotherm of the OM samples was measured on a Micromeritics ASAP 2460 Surface Area and Pore Size Analyzer within a pressure range 0.0001–0.03 at 273 K. The DFT (density functional theory) models were employed to determine the pore structure parameters with CO_2_ adsorption data.

The density of OM was tested on a Micromeritics AccuPyc II 1340d helium pycnometer following the Chinese national standard GB/T217-2008.

#### 3.3.2. FIB-SEM Observation on Shale Sample

In this study, the sample XS-05 was subjected to OM pore morphology observation using a FEI Helios NanoLab 600i in Tsinghua University, Beijing, China. The raw sample was cut into a cylinder with a diameter of 6 mm and a thickness of 15 mm using the wire-cutting equipment. Then, a smooth section was polished on the cylinder by Ar^+^ ion beam, which is used to observe the surface topography, and a region of interest was selected to conduct FE-SEM observation. The sample was cemented on the SEM sturm and coated with gold to make the surface electrically conductive before observation. The distribution of OM in shale is heterogeneous. In order to characterize the OM pore structure in shale, an area rich in OM was selected as the target to be milled. Considering the factors of cost and resolution, the cutting area is 20 μm × 17μm.

During the process of milling and scanning, the Ga^+^ ion beam was maintained perpendicular to the cut surface, while the electron beam was at an angle of 48° from the sample surface. The acceleration voltage for Ga^+^ was 30 kV, and the beam current was 2.5 nA. The sample surface was milled by the ion beam as thin as 20 nm to expose subsurface features, and the electron beam scanned and imaged the subsurface of the sample. By the continuous and repeated milling and imaging, a series of SEM images was obtained with a resolution of 19.5 nm.

#### 3.3.3. TEM Observation on OM Sample

The TEM experiment was employed to identify the OM pores within the shale sample that were less than 10 nm. The TEM images were obtained on a JEM-200F TEM in Taiyuan University of Technology, Taiyuan, China at 200 kV. Before the observation, the fine-powder OM sample was dispersed in ethanol by ultrasonic wave and sprayed over a copper micro-grid.

## 4. Conclusions

In this research, a comprehensive study combined the experimental and computational method to perform an investigation of the molecular structure and micropore structure of OM in Shanxi formation shale, and the methane adsorption capacity of OM was discussed as well.

The results of the elemental analysis show that the OM in the black Shanxi formation shale is rich in carbon and poor in hydrogen, with a small amount of oxygen, indicating the OM belongs to the type III kerogen. The atomic ratios of H/C, O/C, N/C, and S/C are about 0.51, 0.047, 0.012, and 0.009, respectively.

The peak fitting of ^13^CNMR and XPS spectra suggests the aromaticity of OM is 75.57%, and the ratio of aromatic bridge carbon to aromatic peripheral carbon is 0.196, from which it can be inferred that the aromatic structural unit of OM is mainly composed of the naphthalenes. The most oxygen-containing groups are the ether group (C–O), accounting for 60.70%, followed by the carbonyl group (COO-) and carboxyl group (C=O), with proportions of 24.5% and 14.50, respectively. The nitrogen is present mainly in forms of pyridine and pyrrole, and their ratio is about 2.8:1. The sulfur-containing functional groups mainly consist of thiophenes and sulphoxides, with a ratio of 3:2.

With the chemical structural parameter obtained from experiments, the average 2D OM molecular structure was established and defined as C_177_H_160_O_8_N_2_S with a molar weight of 2474. Based on the 2D model, the 3D OM macromolecular model was reconstructed and optimized with the data of the helium density test and low-pressure CO_2_ adsorption experiment. The 3D OM macromolecular model contains 20 2D models with a helium density of 1.41 g/cm^3^. The pores in the 3D model varied from 3 Å to 10 Å, with pores in 3–7 Å being the dominant pores.

The 3D OM micropores model was built with FIB-SEM images and suggests that the OM pore size varied from 20 nm to 350 nm, and the OM pores in TEM were less than 10 nm. Both of them are isolated pores with poor connectivity. Gas diffusion in OM is mainly conducted in pores less than 1 nm.

The methane adsorption simulation results indicate that the methane adsorption capacity of the 3D OM macromolecular model increases with the increase of pressure in the range of 1–4 MPa. When the pressure is more than 4 MPa, the increment of methane adsorption capacity gradually slows down and tends toward saturation. The maximum methane adsorption capacity of the 3D macromolecular model can reach 165.8 cm^3^/g, indicating that OM pores less than 1 nm have a stronger methane adsorption capacity than pores of other sizes.

## Figures and Tables

**Table 1:** Elemental analysis of OM in sample XS-05.

Organic Matter					Atomic Ratio			
C (wt%)	H (wt%)	N (wt%)	S (wt%)	O(wt%)	H/C	O/C	N/C	S/C
87.61	3.71	1.2	1.89	5.51	0.51	0.047	0.012	0.009

**Table 2:** Structure parameters from ^13^CNMR spectra of OM by peak fitting.

Type	Parameters for Aromatic Carbon								Parameters for Aliphatic Carbon			
Parameters	*f_a_*	*f_a_^C^*	*f_a_’*	*f_a_^N^*	*f_a_^H^*	*f_a_^P^*	*f_a_^S^*	*f_a_^B^*	*f_al_*	f_al_^*^	*f_al_^H^*	*f_al_^O^*
Chemical Shift (ppm)	>90	>165	100–165	129–165	100–129	148–165	137–148	129–137	12–90	12–90	22–50	50–90
Value (%)	78.54	2.97	75.57	27.12	48.45	4.57	10.21	12.34	21.46	6.65	10.43	4.38
*X_BP_*	0.195											

Note: *f_a_*, total sp^2^ hybridized carbon; *f_al_*, total sp^3^ hybridized carbon; *f_a_^C^*, carbonyl; *f_a_*, aromatic carbons; *f_a_^H^*, protonated and aromatic carbon; *f_a_^N^*, non-protonated aromatic carbons; *f_a_^P^*, phenolics fraction; *f_a_^S^*, alkylated aromatic carbon; *f_a_^B^*, aromatic bridgehead carbons; f_al_^*^, CH_3_ or quaternary carbons; *f_al_^H^*, CH or CH_2_; *f_al_^O^*, aliphatic carbons bonded to oxygen.

**Table 3:** The XPS data of the OM sample.

Spectrum	Position (eV)	FWHM (eV)	Percentage (%)	Assignment
C 1s	284.8	1.13	68.30	C–C, C–H
	285.2	1.65	16.20	C–O
	286.7	3.35	10.70	C=O
	290.1	3.14	4.80	O–C=O
O 1s	531.2	1.45	14.80	C=O
	532.7	2.18	60.70	C–O
	533.9	1.39	24.50	O–C=O
N 1s	398.5	1.09	66.00	Pyridinic nitrogen
	400.5	1.59	24.00	Pyrrolic nitrogen
	402.5	2.82	10.00	Quaternary nitrogen
S 2p	164.0	0.97	60.50	Thiophenes
	165.2	1.15	39.50	Sulphoxides

**Table 4:** Types of aromatic structure units in the OM single-molecular model.

Type	Number	Type	Number
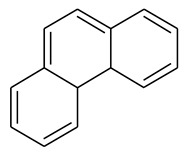	2	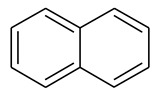	7
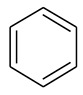	4	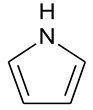	2
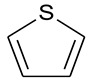	1	*X_BP_* = 0.196	

**Table 5:** TOC of shale samples.

Samples	XS-01	XS-02	XS-03	XS-04	XS-05	XS-06
TOC (%)	4.11	4.77	3.47	3.21	20.3	3.95

## Data Availability

Not applicable.
